# REG-O3 chimeric peptide combining growth hormone and somatostatin sequences improves joint function and prevents cartilage degradation in rat model of traumatic knee osteoarthritis

**DOI:** 10.1371/journal.pone.0231240

**Published:** 2020-04-14

**Authors:** Rodrick Montjean, Sonia Escaich, Raffaello Paolini, Claude Carelli, Sébastien Pirson, Thibaut Neutelings, Yves Henrotin, Christelle Vêtu

**Affiliations:** 1 Regulaxis SAS, Romainville, France; 2 ESE Conseil, Saint-Cloud, France; 3 Artialis SA, Tour GIGA, CHU Sart-Tilman, Liège, Belgium; 4 Bone and Cartilage Research Unit, Arthropôle Liège, University of Liège, Liège, Belgium; 5 Physical Therapy and Rehabilitation Department, Princess Paola Hospital, Vivalia, Marche-en-Famenne, Belgium; Universite de Nantes, FRANCE

## Abstract

**Objective:**

REG-O3 is a 24-aminoacid chimeric peptide combining a sequence derived from growth hormone (GH) and an analog of somatostatin (SST), molecules displaying cartilage repair and anti-inflammatory properties, respectively. This study aimed to investigate the disease-modifying osteoarthritis drug (DMOAD) potential of REG-O3 by analyzing its effect on pain, joint function and structure, upon injection into osteoarthritic rat knee joint.

**Design:**

Osteoarthritis was induced in the right knee of mature male Lewis rats (n = 12/group) by surgical transection of the anterior cruciate ligament (ACLT) combined with partial medial meniscectomy (pMMx). Treatments were administered intra-articularly from fourteen days after surgery through three consecutive injections one week apart. The effect of REG-O3, solubilized in a liposomal solution and injected at either 5, 25 or 50 μg/50 μL, was compared to liposomal (LIP), dexamethasone and hyaluronic acid (HA) solutions. The study endpoints were the pain/function measured once a week throughout the entire study, and the joint structure evaluated eight weeks after surgery using OARSI score.

**Results:**

ACLT/pMMx surgery induced a significant modification of weight bearing in all groups. When compared to liposomal solution, REG-O3 was able to significantly improve weight bearing as efficiently as dexamethasone and HA. REG-O3 (25 μg) was also able to significantly decrease OARSI histological global score as well as degeneration of both cartilage and matrix while the other treatments did not.

**Conclusion:**

This study provides evidence of a remarkable protecting effect of REG-O3 on pain/knee joint function and cartilage/matrix degradation in ACLT/pMMx model of rat osteoarthritis. REG-O3 thus displays an interesting profile as a DMOAD.

## Introduction

Osteoarthritis (OA), one major issue in public health due to the wide proportion of the affected population [1,2], lacks disease-modifying treatment. The commonly used pharmaceutical treatments for OA, including acetaminophen, nonsteroidal anti-inflammatory drugs and intra-articular injection of corticoids and hyaluronic acid (HA), only focus on symptoms [3–7]. Even for these intra-articular (IA) injections, whose symptomatic effects were documented in precise indication, there is no consensus on their potential disease-modifying activities. There is therefore an urgent need to develop an innovative efficient and well tolerated drug able to either prevent cartilage from degradation or stimulate its repair.

Many growth factors and hormones have been studied as putative therapeutic molecules for articular defects due to their crucial role in the control of cartilage homeostasis [8]. Among them, growth hormone (GH) and insulin-like growth factor-1 (IGF-1) received a particular attention for their fundamental role in bone formation through direct effects on osteoblasts and osteocytes [9], in linear and radial bone growth [10] and in cartilage extra-cellular matrix synthesis [11–13]. Recently, a clinical study has shown that adding GH to platelet rich plasma for IA injection improved function of the osteoarthritic knee joint in a short period of time [14].

Somatostatin (SST) is involved in the control of cartilage homeostasis *via* its inhibiting action on GH/IGF-1 axis [15]. Besides its indirect action on cartilage *via* GH/IGF-1 axis, SST is also able to directly modulate cartilage development. SST indeed shows antiproliferative effects on cultured human fetal epiphyseal chondrocytes, as it does in its target cells such as in lymphocytes [16,17].

Clinical trials aiming to test the therapeutic potential of IA injections of SST for rheumatoid arthritis and OA provided very interesting data. Such SST injections reduced progressively joint inflammation in patients with rheumatoid arthritis whereas they improved joint function in patients with knee OA. Interestingly, SST was able to decrease the pain felt by both types of patients [18,19].

REG-O3 is a 24-aminoacid synthetic chimeric peptide combining a short sequence derived from GH (aa G129-L138) and an analogue of SST-14 in which both aminoacids C3 and C14 have been changed into S3 and S14, respectively (patent WO 2010/105685 A). This compound was initially designed to regulate the GH/IGF-1 axis effect on metabolism *in vivo*, to promote the GH-dependent cell growth and to inhibit the anti-proliferative effect of SST. Internal *in vitro* results showed that REG-O3 was able to increase IGF-1 and IGF-1 receptor expression on murine primary chondrocytes, suggesting that REG-O3 may promote cartilage repair (patent WO 2010/105685A https://patentscope.wipo.int/search/en/detail.jsf;jsessionid=62B793EF30CAA20E9E6F564C12EAE2E1.wapp2nA?docId=WO2010105685&tab=PCTBIBLIO).

The present study aimed to assess if REG-O3 was able to improve joint function, reduce pain and preserve articular structure in a rat model of OA induced by the surgical transection of the anterior cruciate ligament (ACLT) combined with the partial medial meniscectomy (pMMx).

In this study, we provide evidence for significant REG-O3-dependent improvement in pain/joint function and a REG-O3 protective effect against cartilage degradation.

## Materials and methods

### Solid-phase synthesis of REG-O3

REG-O3 is a synthetic chimeric peptide combining a short aminoacids sequence derived from the growth hormone (aa G129-L138) and an analogue of active somatostatin (SST-14) in which both aminoacids C3 and C14 have been changed into S3 and S14, respectively. This REG-O3 peptide displays a molecular weight of 2797.37 g/mol and a basic pHi (10.43). The peptide has been fully chemically-produced according to the following steps of solid-phase synthesis with a 15-mmol scale.

The solid-phase peptide synthesis (SPPS) of REG-O3 was engaged with a peptide bond formation between a N^α^-derivatized aminoacids protected by Fmoc group (9-H-Fluoren-9-yl-methoxycarbonyl) and the resin (4-MethylBenzHydrylAmine (MBHA) Resin, 0.70 mmol/g). Then, the SPSS was followed by successive deprotection-coupling cycles of N^α^-derivatized aminoacids. The deprotection and/or coupling cycles are repeated until the desired sequence of aminoacids is generated. The N^α^-protecting group was removed using a Piperidine 25%/DMF (N,N-DiMethylFormamide) solution in order to free the amine group. Once the nascent oligopeptide-linker-support was washed with solvent (DMF), thus removing unreacted material, a quantification of residual piperidine has been performed. This step was repeated until the residual piperidine was lower than 300 ppm. The coupling of the next aminoacids was performed *via* carboxylate activation by using the DIC (N,N′-DiIsopropylCarbodiimide)/HOBt (1-HydrOxyBenzotriazole) (2.5 eq). The correct coupling was monitored with suitable in-process control (*IPC*) tests such as Kaiser test; for some aminoacids, double and triple couplings were conducted with DIC/HOBt and PyBOP (benzotriazol-1-yl-oxytripyrrolidinophosphonium hexafluorophosphate)/HOBt/DIEA (N,NDiIsopropylEthylAmine), respectively. 69.6 g of REG-O3 peptide attached to the resin (Fmoc-REG-O3-Mppa-MBHA-resin) were obtained with a yield of 89%.

Upon deprotection the crude peptide was acetylated by solubilization in an acetic acid/water/acetonitrile solution (20/20/60; v/v/v) at 10 g/L for 3h. Peptide cleavage from the resin and side-chain deprotection were achieved by treatment with a TFA (TriFluoroacetic Acid)/TIS (TriIsopropylSilane)/water solution (95:2.5:2.5 v/v/v, 1 mL/100 mg of resin-bound peptide). The crude peptide was precipitated from the cleavage mixture by addition of ice-cold Et_2_O, centrifuged, washed with ice-cold Et_2_O (x3), dried and lyophilized. The crude peptide was purified using a preparative RP-HPLC (Reverse Phase-High Performance Liquid Chromatography) system, with a C18 column from Phenomenex (100 Å 15 μm, 250*50 mm–reference 00G-4273-V0-AX) working at 93 mL/min, a solvent system containing Buffer A: 0.1% TFA/water—Buffer B: 0.1% TFA /water/acetonitrile and a gradient of 25 to 53% of buffer B in 24 min.

The semi pure peptide obtained above was finally purified (5 g per injection) using an ion exchange RP-HPLC system with a C18 column from Phenomenex (100 Å 15 μm, 250*50 mm–reference 00G-4273-V0-AX) working at 93 mL/min using as solvent system Buffer A: 0.1% Ac-OH/water—Buffer B: 0.1% Ac-OH/water/acetonitrile and a gradient of 0 to 100% of buffer B in 25 min.

The final peptide purity was checked by HPLC. We obtained 9.8 g of pure REG-O3 peptide with 97.38% purity.

### Experimental model and study groups

Mature male Lewis rats (n = 72; Charles River Laboratories. Ecully, France) were housed, by two, in ventilated cages (Sealsafe® Plus GR900. Leicester, UK) in compliance with the European Directive ETS 123 and under controlled temperature (between 20°C and 24°C), humidity (between 45% and 65%) and light cycles (light is on between 7:30 AM and 7:30 PM). Rats were allowed food and water *ad libitum*. The protocol was approved by the Ethical Committee of the “Centre d’Economie Rurale” (CER) Marloie (Registration Number: PS-2017-NOV-0) and the study conducted by Artialis SA (Liège, Belgium).

OA in the right knee was surgically induced in all rats by anterior cruciate ligament transection (ACLT) combined with partial medial meniscectomy (pMMx) [20–22]. Of note, rats were 12 weeks old and weighing approximately 380 g at the time of surgery (T0 where T means time point corresponding to weeks after surgery). Special attention was taken in order to minimize animal suffering and distress. Rats received buprenorphine (0.01–0.05 mg/kg, subcutaneous) before anesthesia and surgery. Anesthesia was induced by 3–4% isoflurane/O2 and maintained during the entire surgery procedure using a mask with 2% isoflurane/O2 mixture. Before wound closure a peri-articular injection of bupivacaine 0.5% was performed. Animals received sterile physiological serum (500 μL subcutaneous) right after surgery, meloxicam (subcutaneous) one day after surgery, analgesic treatment with buprenorphine immediately after surgery and maintained during 72h. When needed, skin wound was treated once a day with silver sulfadiazine topical ointment (Flamazine).

**Fig 1:**
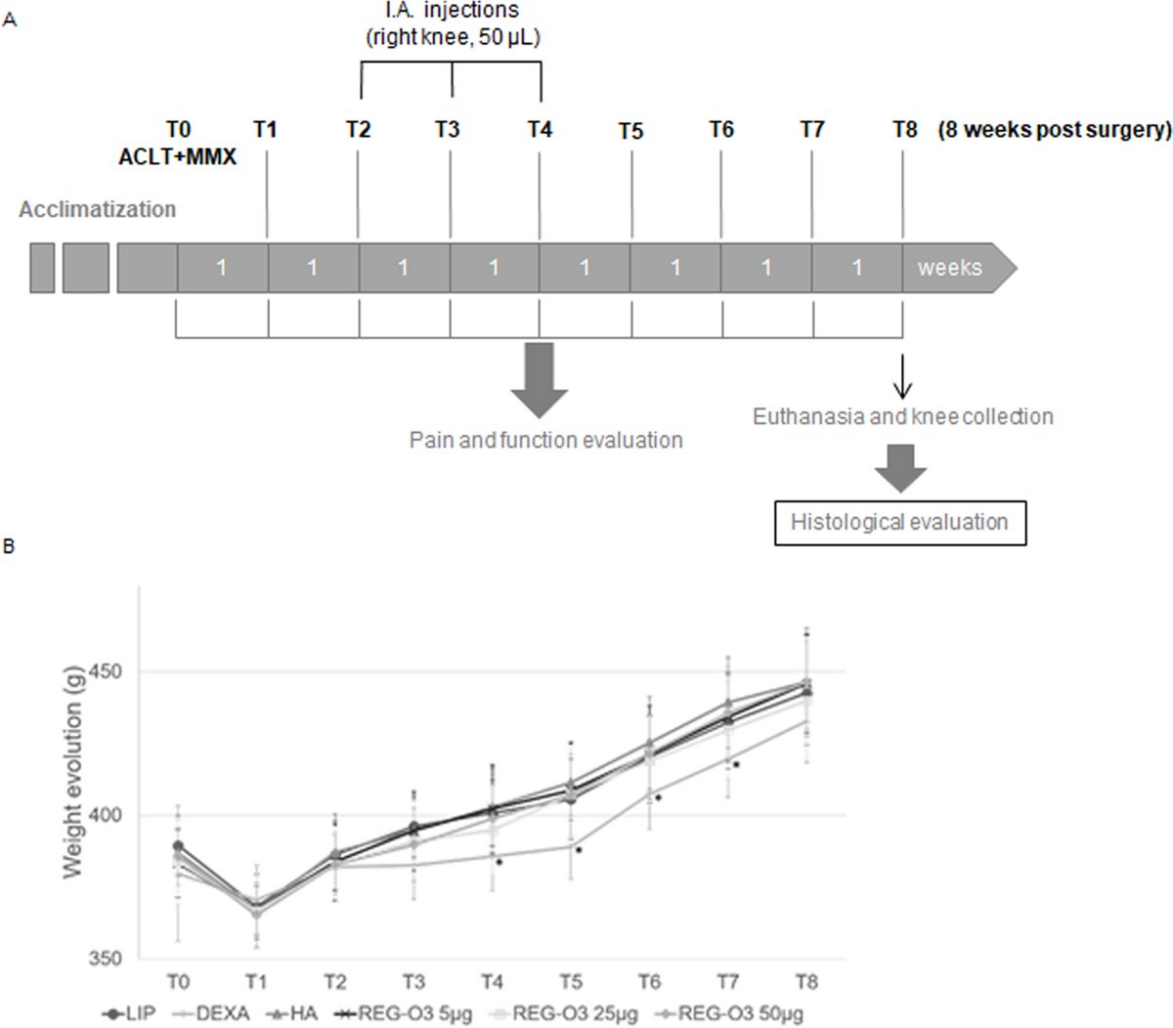
Study design and bodyweight evolution. (A) Knee osteoarthritis was induced through the surgical anterior cruciate ligament transection (ACLT) combined with partial medial meniscectomy (pMMx) of the right knee at T0. Treatments with reference and test items were initiated 14 days after surgery (ACLT/pMMx) and administered through three intra-articular injections of 50 μL in the right operated knee, three times, one week apart (T2, T3 and T4; T: Time point). Pain and joint function were monitored during the entire in-life phase once a week from T0 to T8 (8 weeks after surgery). Animals were euthanized 8 weeks after the surgery (T8) with a subcutaneous injection of a ketamin/medetomidine mix and further exsanguination. The knees were collected and processed for histological evaluation. (B) Mean body weight evolution (in g) in all groups for the entire duration of the in-life phase. With longitudinal analysis model for each group at each time point, animals treated with dexamethasone (DEXA) showed a significant difference in their body weight when compared to the rats that received hyaluronic acid (HA) injections (from T4 to T7), to those injected with 5 μg of REG-O3 (T4 and T5), 25 μg of REG-O3 (T5) and 50 μg of REG-O3 (T5).

Each animal then received one intra-articular injection, into the right knee, at days 14 (T2: 2 weeks after surgery), 21 (T3: 3 weeks after surgery) and 28 (T4: 4 weeks after surgery) under anesthesia in full aseptic conditions. These mature male Lewis rats were distributed by twelve into six experimental groups: 1) negative control group received the placebo liposomal solution (vehicle; LIP); 2) first positive control group received dexamethasone (DEXA) (Aacidexam® 0.5 mg/kg [23]; Oss, the Netherlands); 3) second positive control group received hyaluronic acid (HA) 1 mg/kg [24,25] (50 μL from a stock solution at 10 mg/mL); HTL Biotechnology, France); 4–6) animals from these groups received REG-O3 mixed in liposomal solution and injected at the quantity of 5 μg, 25 μg or 50 μg corresponding to final concentrations of 10^−5^ M, 5.10^−5^ M, or 10^−4^ M, in the knee joint, respectively. Items were administered with a 26G needle (except 21G for HA) under a volume of 50 μL at each injection. General appearance, viability, mobility and stools were monitored daily for all rats for the entire duration of the study. In addition a close monitoring of the animals was performed for the 3 days after surgery and for the first three days following the injection in order to record any sign of acute reaction and animal suffering. The following parameters were recorded: general appearance and appearance of the application site, natural behavior, knee swelling, hunching and convulsion as well as food and water intake. All parameters were scored in order to identify any sign of distress or suffering during the study. Animals were followed eight weeks after knee surgery (Fig 1A).

### Function and pain assessment

Pain and function were assessed through the weight bearing of animals using the Dynamic Weight Bearing test (DWB, Bioseb, France) [26]. It consists in an operator-independent system that evaluates the weight distribution of each limb by a sensor made of 2000 independent load cells of very high sensitivity. It gives a measure of the postural deficit when the animal is moving spontaneously during a five-minute experiment. All animals were monitored on the DWB system once a week from T0 (before surgery, reference time point) to T8 (eight weeks after surgery and before euthanasia). The rear left/rear right paw (rear L/R) ratio weight bearing as well as the operated rear right weight bearing were extracted from the acquired data.

### Histological evaluation of articular cartilage and synovial membrane

Whole knee joints were fixed in 4% paraformaldehyde (PFA) immediately after dissection and were processed as recommended by Gerwin et *al* [20]. Frontal histological sections (5 μm) were performed with a standard microtome on paraffin-embedded samples after appropriate decalcification (DC2 decalcifier, QPath). Three sections 200 μm apart were performed in weight bearing area and stained with Safranin-O and Fast green. An additional slide was stained with Toluidine blue. The histological analysis of the entire knee (evaluation of the 4 compartments) was performed in accordance with the OARSI (OsteoArthritis Research Society International) recommendations [20] by the evaluation of the following parameters: cartilage matrix loss width (score 0–5 for superficial, intermediate and deep zones of cartilage; 3 values for each compartment); cartilage degeneration score (score 0–5 for outer third, middle third and inner third of cartilage; 3 values for each compartment); significant cartilage degradation (representing the loss of more than 50% of the tissue) width (score 0–5); total cartilage degeneration width (corresponding to any kind of cartilage modification) (score 0–5); calcified cartilage and subchondral bone score (0–5); osteophyte score (score 0–4); synovial membrane reaction (score 0–4). Each slide was scored under a light microscope by two trained experts (blinded for the treatment groups), and the final score consists in the mean of the score obtained for each evaluated parameter. No actual measures were performed on the histological sections. All widths were estimated as follows with a semi-quantitative score: 0 = no modified tissue, 1 = modification concerning less than 10% of the total width; 2 = modification concerning 10–25%; 3 = modification concerning 25–50%; 4 = modification concerning 50–75%; and 5 = modification expanding over 75% of the total width of the concerned tissue. The histological global score is the result of the sum of all the mean final scores obtained for the four compartments of the knee joint (total score 0–200).

### Statistical analysis

The distribution of the quantitative data was investigated. Non-parametric tests were used in case of non-normality of the distributions.

For DWB results (using SAS Version 9.4), the time evolution across the eight time points was investigated. To this aim, a mixed model was fitted to the data to test. The covariates included in the model was the time (considered as a qualitative variable, “T0” was considered as the reference) and an interaction term with the group indicator (“LIP”, “DEXA” or “HA” were as the group reference). This statistical method allowed the comparison of response curves between treatments while accounting for repeated data within individuals. This statistical approach was applied to test for time differences in each group, as well as for group differences at each time point. Data are presented as mean and 95%CI. Considering the number of comparisons performed for each data set, the significance level was calculated at 0.0003.

For the histological data analysis (using GraphPad Prism version 8), parametric test was applied to normally distributed data (One-way ANOVA test followed, if positive, by Dunnett’s multiple comparison test). Parametric data are presented as mean and 95%CI and individual points are presented on the graph. When non-normality was observed, non-parametric test was applied (Kruskal-Wallis test followed, if positive, by Dunn’s multiple comparison test). Non-parametric data are presented as median and interquartile range (Q1–Q3) and individual points are presented on the graph. The significance level was considered below 0.05.

## Results

### Product tolerability and clinical observation

Before being injected in rats, the cytotoxic and genotoxic profile of REG-O3 peptide was evaluated. In this frame, the cytokinesis-block proliferation index (CBPI) quantification, replication index (RI) and micronuclei induction in V79-4 cell lines grown were observed with increasing concentrations of REG-O3 (S1 Appendix, method). Thus, it was showed that REG-O3 induced neither *in vitro* cytotoxic (S1 Fig in S1 Appendix) nor *in vitro* genotoxic effects (S2 Fig in S1 Appendix). Furthermore, even if REG-O3 is a peptide that can be highly exposed to proteases-linked degradation within biological samples including synovial fluid, it was also demonstrated that REG-O3 displays a 24h stability in synovial fluid (S3 Fig, S2 Appendix). REG-O3 was therefore intra-articularly injected knowing that it will not induce any negative effects on the cells present in the knee joint and will display an extensive stability *in situ*.

The mean weight evolution of rats was followed for the entire in-life phase duration (Fig 1B). The curves showed a loss of body weight one week after the surgery followed by a constant weight gain for all animals until the end of the study except with dexamethasone (DEXA) from T2 to T5 (corresponding to the time of treatment administration). With longitudinal analysis model for each group at each time point, animals treated with DEXA showed a significant difference in their body weight when compared to the rats that received the hyaluronic acid (HA) injections (from T4 to T7), to those injected with 5 μg of REG-O3 (T4 and T5), 25 μg of REG-O3 (T5) and 50 μg of REG-O3 (T5) (Fig 1B).

The natural behavior, the appearance of the rats, food and water intake were normal for all rats after either test or reference items injections. No signs of toxicity linked to the tested compound were observed in any groups based on the evaluated parameters.

No local inflammatory reaction or joint swelling were observed at the injection site whatever the treatment administered.

### Evaluation of the effect of REG-O3 on pain and function evaluated by DWB

**Fig 2:**
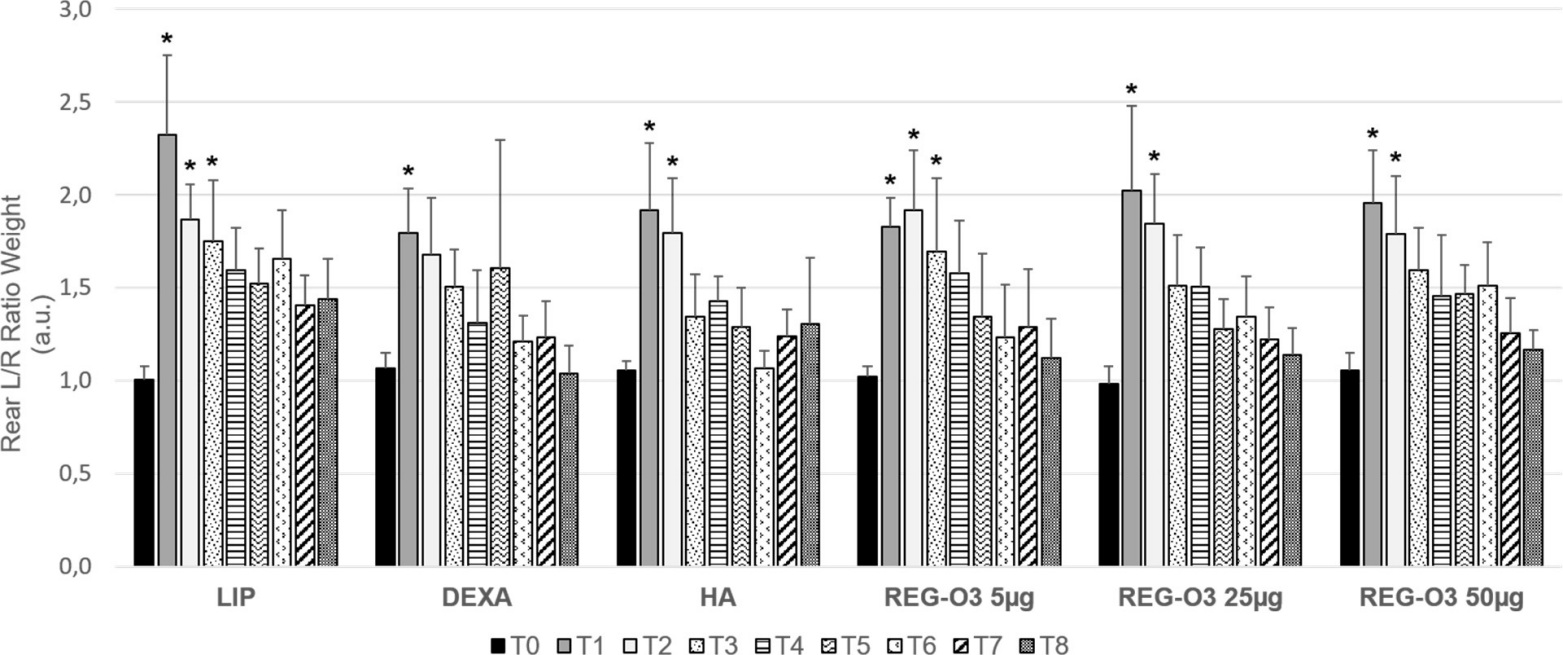
Evolution of rear pain/joint function measured by dynamic weight bearing test. Evolution of rear left/right ratio (rear L/R) for weight bearing over time in all groups (n = 12/group). Results are presented as mean ± 95% CI at each time point (T corresponding to weeks after surgery) for each group (longitudinal analysis model for each group at each time point, *: p-value ≤0.0003 versus T0 was considered as significant).

The rear left/rear right (rear L/R) ratio corresponded to the weight distribution between rear left (non-operated) and rear right (operated) limb (Fig 2). Animals that received the vehicle (liposomal solution, LIP) displayed a significant 2.4-fold increase in rear L/R weight ratio (Fig 2), one week after surgery (T1). These results underline that these animals bore more on the rear left paw (non-operated) resulting from pain/discomfort on the operated right side. The animals treated either with positive control items regarding pain (dexamethasone (DEXA), hyaluronic acid (HA)) or with REG-O3 behaved similarly to the vehicle-treated ones. Indeed, the rear L/R ratio for weight bearing was also significantly increased after surgery for all those study groups at T1 (Fig 2).

The vehicle-treated (LIP) animals still bore more on the rear left paw until 3 weeks after the surgery (T3) since they presented a weight bearing that was not significantly different from the one displayed at T0, only 2 weeks after the first IA injection of placebo (T4). In contrast, DEXA and HA-treated animals recovered earlier. Indeed, while DEXA-treated rats exhibited a weight bearing that was not significantly different from the one displayed before the first IA injection at T2, the HA-treated ones did 1 week after the first IA injection (T3) (Fig 2). Like HA-treated rats, animals that received either 25 μg or 50 μg of REG-O3 recovered 1 week after the first injection (T3) (Fig 2). In contrast, similarly to the vehicle-treated animals, rats treated with 5 μg of REG-O3 recovered 2 weeks after the first injection (T4) (Fig 2).

**Fig 3:**
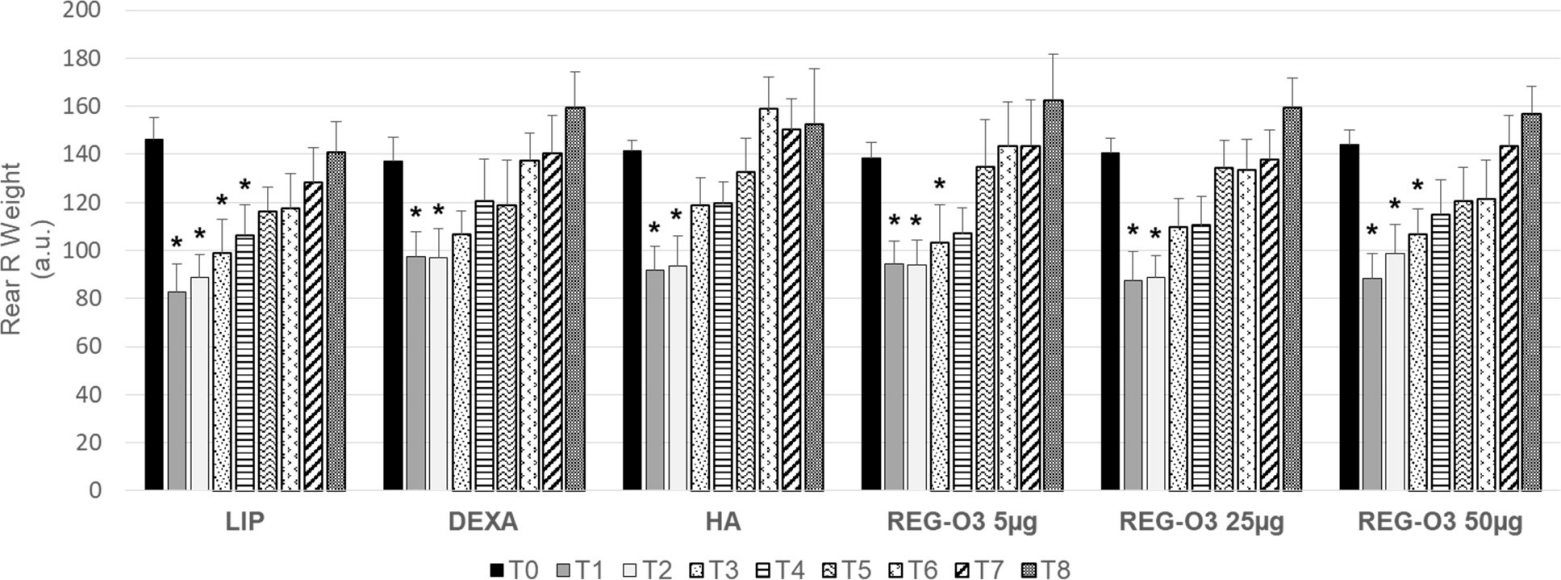
Evolution of the operated rear right pain/joint function measured by dynamic weight bearing test. Evolution of rear right (R) weight bearing over time in all groups (n = 12/group). Results are presented as mean ± 95%CI at each time point (T corresponding to weeks after surgery) for each group (longitudinal analysis model for each group at each time point, *: p-value ≤0.0003 versus T0 was considered as significant).

To observe the direct consequence of the ACLT/pMMx surgery, the weight bearing was directly assessed on the operated rear right limb (Fig 3). In vehicle-treated group (LIP), the weight bearing on the rear right paw (operated) drastically decreased one week after the surgery (T1). These animals indeed exhibited a significant 1.8-fold decrease of rear right weight ratio (Fig 3). Interestingly, the animals treated either with positive control items (DEXA, HA) or with REG-O3 behaved similarly to the vehicle-treated rats one week after the surgery (T1). Indeed, at this time point, the rear right weight (R) bearing was significantly decreased after surgery for all those study groups (Fig 3).

The vehicle-treated (LIP) animals presented a weight bearing that was not significantly different from the one displayed at T0 only 3 weeks after the first IA injection of placebo (T5) whereas rats injected with either of the positive control items totally recovered 2 weeks earlier, at T3 (1 week after the first injection) (Fig 3). Like positive controls-treated animals, IA-injected rats with 25 μg of REG-O3 recovered 1 week after the first injection (T3) whereas animals treated with either 5 μg or 50 μg of REG-O3 recovered one week later, i.e. one week earlier than the vehicle-treated rats (T4) (Fig 3).

In total, the evaluation of pain and joint function by DWB shed light on REG-O3 protective effect which was similar to positive control items such as HA, especially when we injected 25 μg of REG-O3.

### Evaluation of the effect of REG-O3 on articular structures by histological analysis

**Fig 4:**
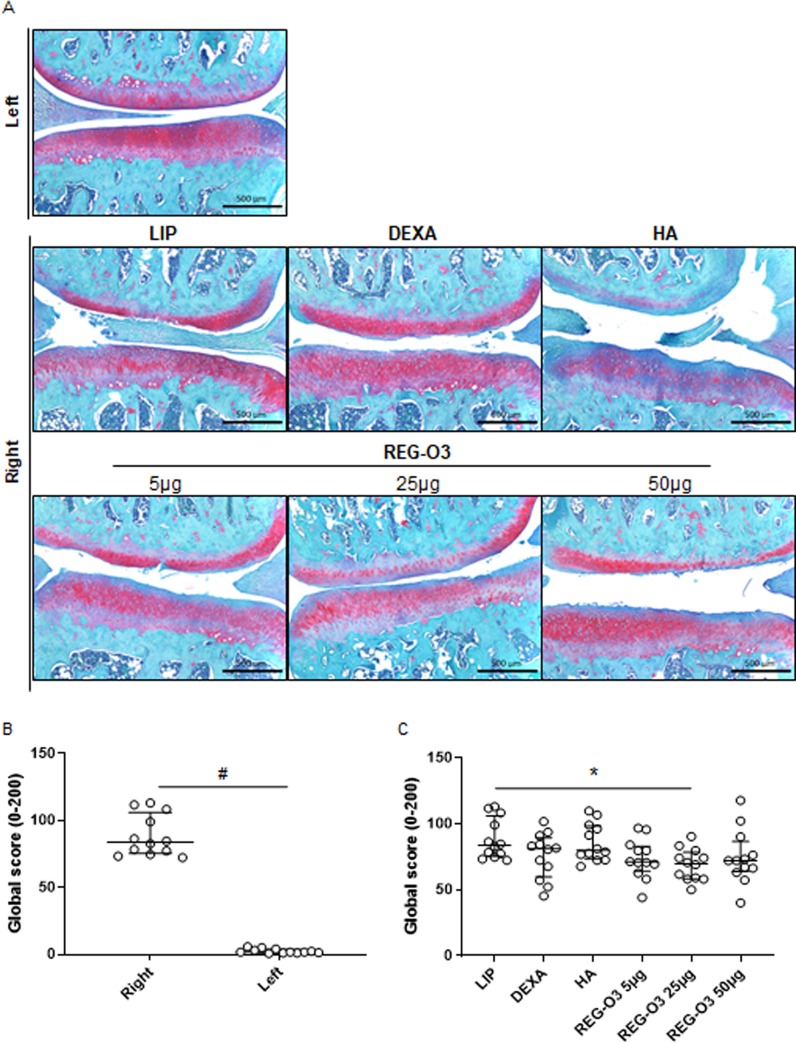
Histological quantification of the OA lesions: OARSI histological global score. (A) The knees of each animal were stained with Safranin O/Fast Green and this panel shows representative section from the medial compartment of the left knee from a placebo-treated rat (LIP) (upper panel) and of the right knees from each study group (middle and lower panels) (5-μm thick section stained with Safranin-O and Fast green). All evaluated slides were taken in the weight bearing zone of the knee joint and the images correspond to the medial compartment. Scale bar: 500 μm (B) Comparison of OARSI score between the right and left knees of the vehicle group (LIP). Data were compared using a Mann-Whitney U-test for non-parametric values (#: p<0.0001 was considered as significant). (C) Global score obtained for the right operated knee from all study groups. Each open dot represents the OARSI histological global score for one rat. Groups were compared to LIP-treated animals using non-parametric Kruskal-Wallis test. Since positive (p = 0.0225), it was followed by multiple comparison Dunn’s test (*: p<0.05 versus vehicle-treated group was considered as significant). Data are presented as median (horizontal bar) and interquartile range (Q1–Q3; vertical bars). Open dots correspond to individual values.

Both knees of each animal were scored using the established OARSI score for rat histopathological evaluation [20]. The OARSI global score gave an overview of the modifications that affects cartilage, calcified cartilage/subchondral bone and synovial membrane (Fig 4). The model validity was first assessed by comparing the global scores for the operated rear right knees to the contralateral non-operated left ones. This comparison revealed that the rear right knees were the site of moderate OA (OARSI global score for right knees, median (Q1–Q3): 83.67 (75.33–106.00) whereas left knees remained normal (OARSI global score for left knees, median (Q1–Q3): 1.67 (1.08–3.75)). This comparison is shown here for the vehicle-treated rats (LIP) on Fig 4A (upper and middle panels) and 4B. We found neither significant difference in non-operated left knees global scores between the study groups nor modifications of the synovial membrane (results presented in S1 Fig in S1 Appendix, S1 Fig).

The knees of each animal were stained with Safranin O/Fast Green and Fig 4A shows representative images of cartilage damage for each group on medial tibial plateaus. When considering the global histological OARSI score, it appeared that the animals treated with 25 μg of REG-O3 displayed a significant decrease of this score when compared to the vehicle-treated ones (LIP) (global OARSI score, median (Q1–Q3): 83.67 (75.33–106.00) for vehicle-treated rats vs. 69.83 (58.25–78.25) for animals treated with 25 μg of REG-O3, p<0.05; Fig 4C). In contrast, neither reference items (DEXA and HA) nor REG-O3, at either 5 μg or 50 μg exhibited such a decrease (Fig 4C). For more specific information about REG-O3 protective effects, we next studied each item of the global histological OARSI score separately i.e. cartilage matrix loss width, cartilage degeneration score, significant cartilage degradation, total cartilage degeneration width, calcified cartilage and subchondral bone, osteophyte score, and synovial membrane reaction.

**Fig 5:**
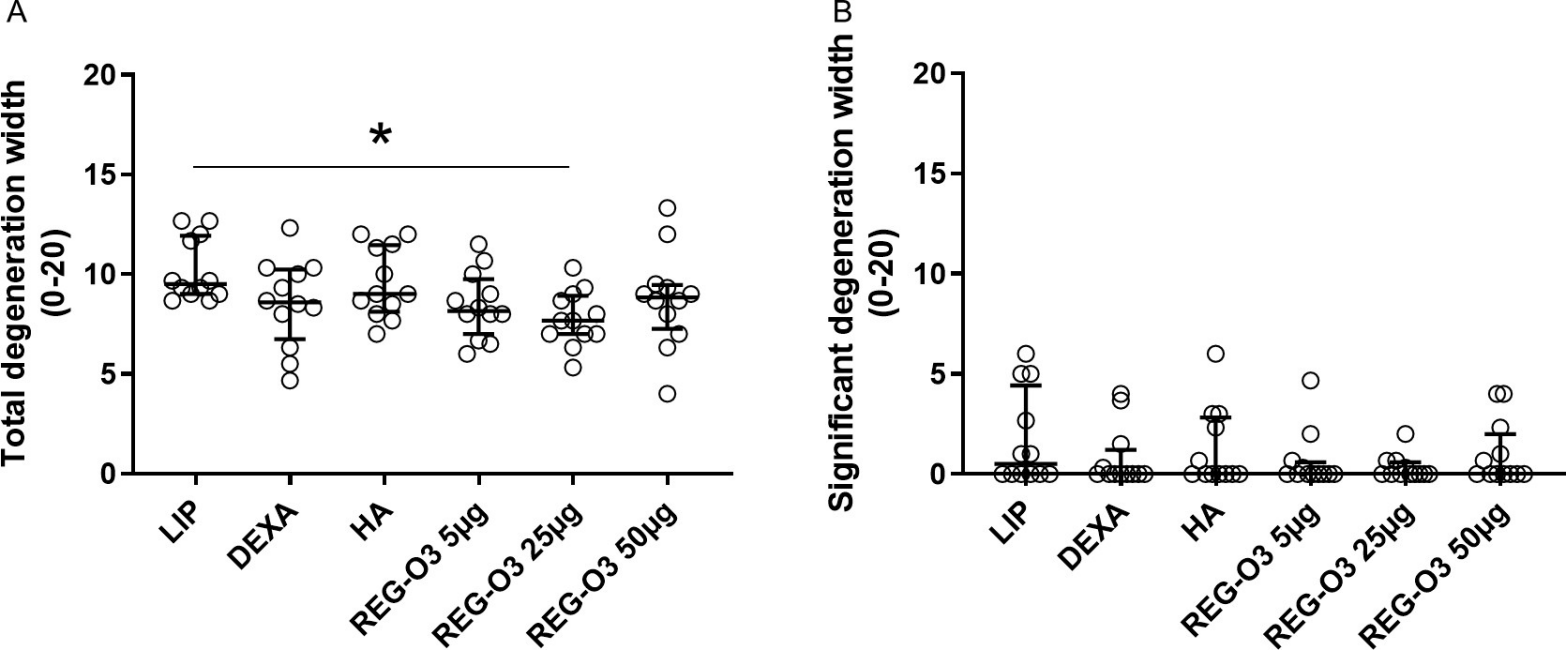
Cartilage degeneration width score. (A) Total cartilage degeneration width for right knees from all study groups. Each dot represents the total cartilage degeneration width for one rat. Groups were compared to vehicle-treated rats (LIP) using non-parametric Kruskal-Wallis test. Since positive (p = 0.0241), it was followed by Dunn’s multiple comparisons test (*: p<0.05 versus LIP was considered as significant). (B) Significant cartilage degeneration width for right knees from all study groups. Each dot represents the significant degeneration width score for one rat. Groups were compared to LIP animals using non-parametric Kruskal-Wallis test which appeared negative (p = 0.7173). Data are presented as median (horizontal bar) and interquartile range (Q1–Q3; vertical bars). Open dots correspond to individual values.

When analyzing total cartilage degeneration width that describes the overall width concerned by cartilage degeneration and considering the entire compartment, we observed that the rats receiving 25 μg of REG-O3 also exhibited a significant decrease of this score when compared to the vehicle-treated ones (LIP) (total cartilage degeneration width, median (Q1–Q3): 9.50 (9.00–11.92) for vehicle-treated animals vs. 7.67 (7.00–8.92) for rats injected with 25 μg of REG-O3, Fig 5A). Of note, neither the treatment with the other tested doses of REG-O3 nor with the reference items induced similar decrease (Fig 5A).

The significant cartilage degeneration width, parameter considering the loss of more than 50% of cartilage thickness in each compartment, has also been assessed in all study groups. However, no significant differences were observed with this significant cartilage degeneration width compared to vehicle-injected rats (LIP) (Fig 5B).

**Fig 6:**
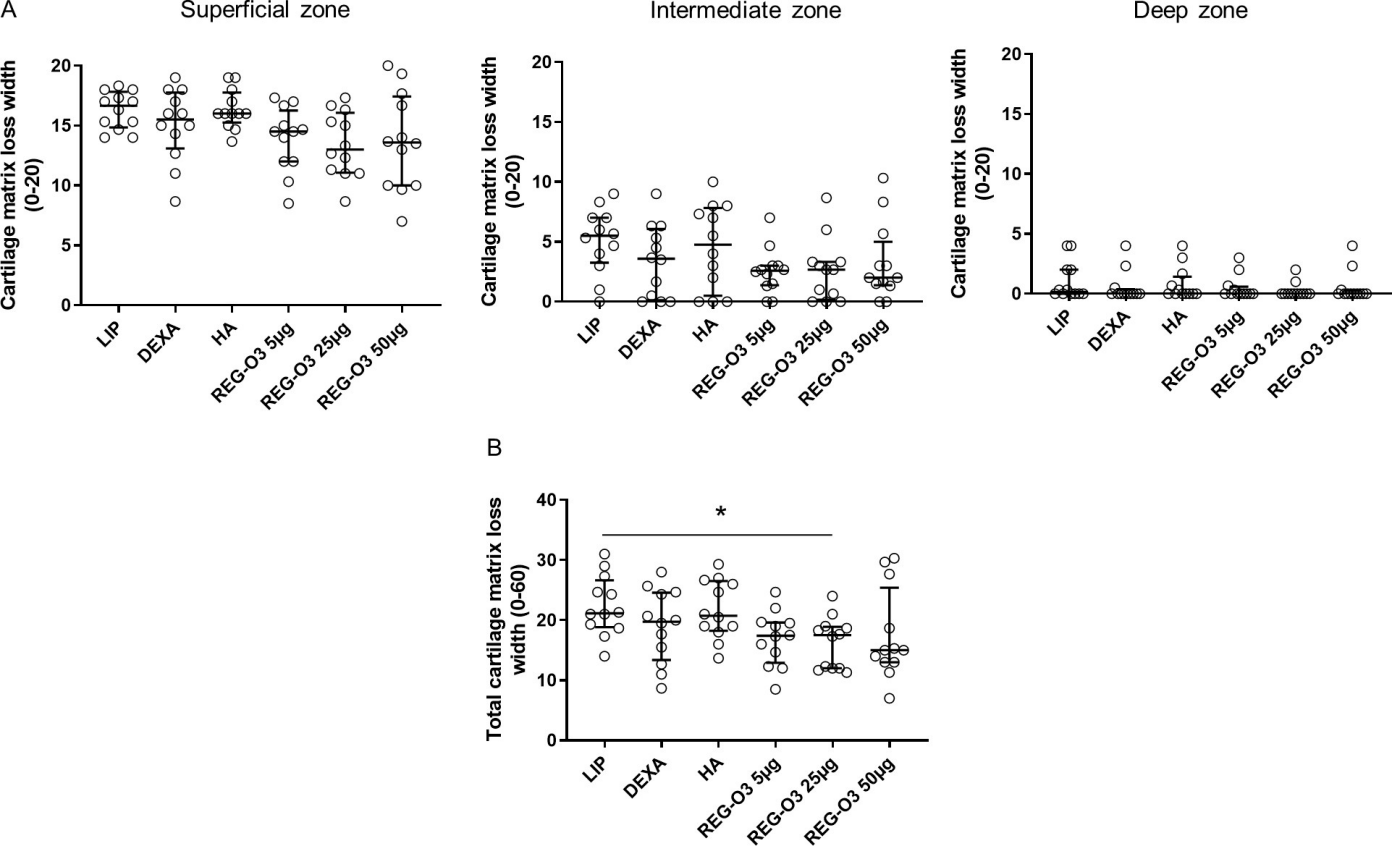
Cartilage matrix loss width score. (A) Cartilage matrix loss width for right knees from all study groups in superficial (left panel), intermediate (middle panel) and deep (right panel) zones. On each graph, each open dot represents the cartilage matrix loss width for one rat. Groups were compared to LIP-treated rats (vehicle) using non-parametric Kruskal-Wallis test (superficial zone: p = 0.0348 since positive followed by Dunn’s multiple comparisons test which turned negative, intermediate zone: p = 0.2212, deep zone: p = 0.5700). (B) Total cartilage matrix loss width for right knees from all study groups. Each dot represents the total cartilage matrix loss width for one animal. Groups were compared to LIP-treated animals using non-parametric Kruskal-Wallis test. Since positive (p = 0.0241), it was followed by Dunn’s multiple comparisons test (*: p<0.05 versus LIP-treated group was considered as significant). Data are presented as median (horizontal bar) and interquartile range (Q1–Q3; vertical bars). Open dots correspond to individual values.

The loss of cartilage matrix in the different zones of cartilage (superficial, intermediate and deep zones) has been studied by quantifying the cartilage matrix loss width (Fig 6A). Whatever the analyzed zone, when considered separately, none of the animals treated with either reference or REG-O3 items showed any significant difference in the cartilage matrix loss width compared to vehicle-injected rats (LIP) (even if Kruskal-Wallis test was positive for the superficial zone, p = 0.0348). Interestingly, when considering the cartilage matrix loss width for the entire cartilage (the three zones pooled together) (Fig 6B), the animals that received 25 μg of REG-O3 displayed a significant reduction of the cartilage matrix loss width comparatively to rats treated with the vehicle (LIP) (cartilage matrix loss width, median (Q1-Q3): 21.17 (18.83–26.67) for vehicle-treated animals vs. 17.50 (12.00–18.92) for rats injected with 25 μg of REG-O3) (Fig 6B). Again, neither the treatment with the other tested doses of REG-O3 nor with the reference items induced a similar decrease in the cartilage matrix loss width for the entire cartilage (Fig 6B).

**Fig 7:**
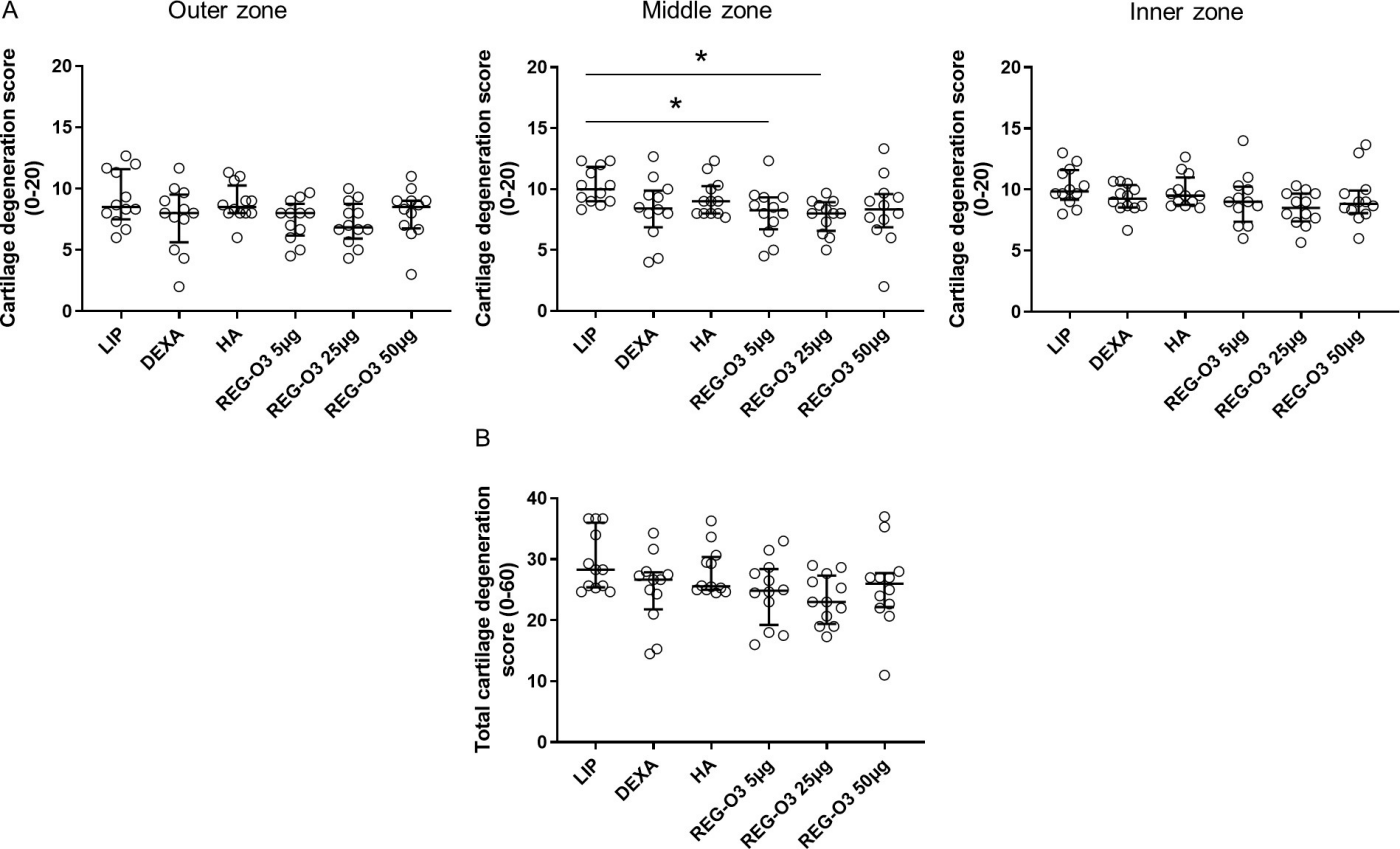
Quantification of cartilage degeneration score. (A) Cartilage degeneration score for right knees from all study groups in outer third (left panel), middle third (middle panel) and inner third (right panel) zones. On each graph, each dot represents the cartilage degeneration score for one rat. The horizontal bar depicts the median values and the vertical bars the interquartile range (Q1–Q3). Groups were compared to LIP-treated rats (vehicle) using non-parametric Kruskal-Wallis test which appeared negative for outer and inner third zones (outer zone: p = 0.1644, inner zone: p = 0.1571). Kruskal-Wallis test, which was positive for middle third cartilage zone (p = 0.0247), was then followed by Dunn’s multiple comparisons test (*: p<0.05 versus LIP-treated group was considered as significant). (B) Total cartilage degeneration score for right knees from all study groups. Each dot represents the total cartilage degeneration score for one animal. The horizontal bar depicts the median values and the vertical bars the interquartile range (Q1–Q3). Groups were compared to LIP-treated animals using non-parametric Kruskal-Wallis test (p = 0.0803).

Finally, we assessed the last parameter allowing the evaluation of cartilage degeneration namely the cartilage degeneration score. This parameter considers any kind of cartilage degeneration, i.e. irregularities, matrix staining, cellularity or matrix loss in the outer third, middle third and inner third zones of each compartment. When compared to vehicle-treated (LIP) rats, none of the animals treated with the test items showed any significant difference in the cartilage degeneration score in both outer and inner zones of cartilage (Fig 7A, left and right panels). Interestingly, the cartilage degeneration score in the middle zone of cartilage was found significantly decreased in animals that received IA injections of REG-O3 at either 5 μg or 25 μg comparatively to the vehicle group (cartilage degeneration score in the middle zone, median (Q1-Q3): 10.00 (9.00–11.83) for negative control group vs. 8.25 (6.71–9.33) for rats treated with 5 μg of REG-O3 and 8.00 (6.58–8.92) for animals that received 25 μg of REG-O3) (Fig 7A, middle panel). In contrast, the animals treated with either the reference items or 50 μg of REG-O3 did not display that significant decrease.

We also considered the cartilage degeneration score for the entire cartilage (outer, middle and inner third zones pooled together) (Fig 7B) but none of the animals treated with the test items showed any significant difference when compared to vehicle-treated rats (LIP) (Fig 7B).

The effects of REG-O3 on articular structures by histological analysis have also been assessed on osteophyte, calcified cartilage/subchondral bone and synovial membrane reaction scores. In these experimental conditions, IA injections of REG-O3 did not induce any significant effect on these read-outs (results presented in S2 Fig in Appendix, S2 Fig).

## Discussion

REG-O3 is a 24-aminoacid synthetic chimeric peptide designed to regulate the Growth Hormone/Insulin like Growth Factor 1 (GH/IGF-1) and the somatostatin (SST)-dependent pathways. Given the known positive effects of both GH/IGF-1 axis and SST on cartilage repair, pain and function in the knee, we hypothesized that REG-O3 could be a disease-modifying osteoarthritis drug (DMOAD) for knee osteoarthritis (OA). The aim of this study was therefore to provide first evidence of REG-O3 disease-modifying effect on knee surgically-induced OA animal model obtained by transection of the anterior cruciate ligament (ACLT) combined with partial medial meniscectomy (pMMx).

This model induced painful OA associated with a decrease in the operated hind limb weight bearing. Therefore, an increase of this operated hind limb weight bearing can be interpreted as resulting from decreased pain and improved joint function. In this study, whatever the dose intra-articularly injected, REG-O3 significantly enhanced the weight bearing of the operated rear right paw indicating that this peptide decreases post-surgical pain. However, depending on the administered dose, the improvement appeared at different times. When injected at either 5 μg or 50 μg (corresponding to final concentrations of 10^−5^ M or 10^−4^ M in the knee joint, respectively), REG-O3 significantly increased weight bearing of the operated rear right paw from two weeks after the first IA injection (four weeks after the surgery) while the vehicle-treated animals displayed a significant enhancement of weight bearing of the operated rear right paw three weeks after the first IA injection (five weeks after the surgery). By contrast, injections of 25 μg of REG-O3 (corresponding to a final concentration of 5.10^−5^ M in the knee joint) led to a significant enhancement of this paw weight bearing one week earlier (one week after the first IA injection i.e. three weeks after the surgery). Interestingly, the recovery of the operated hind limb weight bearing at 25 μg of REG-O3 was similar to that obtained with dexamethasone (DEXA) and hyaluronic acid (HA). These data evidenced an antalgic effect of REG-O3 which, in this model, was comparable to DEXA and HA, two reference drugs commonly used in human clinical practice especially in OA treatment [27]. This effect could be related to the presence of the SST fragment since it was reported that SST exerts an antalgic and anti-inflammatory effect in rheumatoid arthritis (RA) and knee OA [18,19]. Although SST exerts its biological effects *via* specific receptors binding [28], in some cases, SST administration does not display a classical dose-response effect. Indeed, intra-articular injections of SST in the knee of rabbits with chronic arthritis at either 500 μg or 750 μg reduced the signs of arthritis whereas intra-articular injections of SST at 1000 μg did not [29]. In total, the lack of dose-response effect of REG-O3 is not surprising given this SST property and the almost immediate protective effect of REG-O3 on pain/joint function.

This ACLT/pMMx rat model also generated moderate OA according to the histological criteria of OARSI score. Four weeks after the last IA injection (eight weeks after surgery), a putative decrease of OARSI degeneration scores in the operated knee can be interpreted as the result of cartilage degradation prevention.

When 5 μg were intra-articularly injected, REG-O3 significantly decreased the cartilage degeneration score in the middle zone of cartilage in the operated rear right paw. Administration of 25 μg of REG-O3 led to a similar decrease of the cartilage degeneration score in the middle zone of cartilage associated with a significant decrease of OARSI histological global score, cartilage degeneration width and cartilage matrix loss. However, when 50 μg were injected, REG-O3 was unable to significantly modify any of the scores regarding cartilage degeneration. As expected, both DEXA and HA failed to protect cartilage from degeneration since they are clearly known for pain relief rather than protecting cartilage from degeneration [30]. In conclusion, regarding cartilage degeneration, we were able to show that 25 μg of REG-O3 is an efficient dose. Moreover, we observed an absence of REG-O3 dose-response effect between 25 μg and 50 μg suggesting no additional benefits of using 50 μg of REG-O3 which may be confusing.

**Fig 8:**
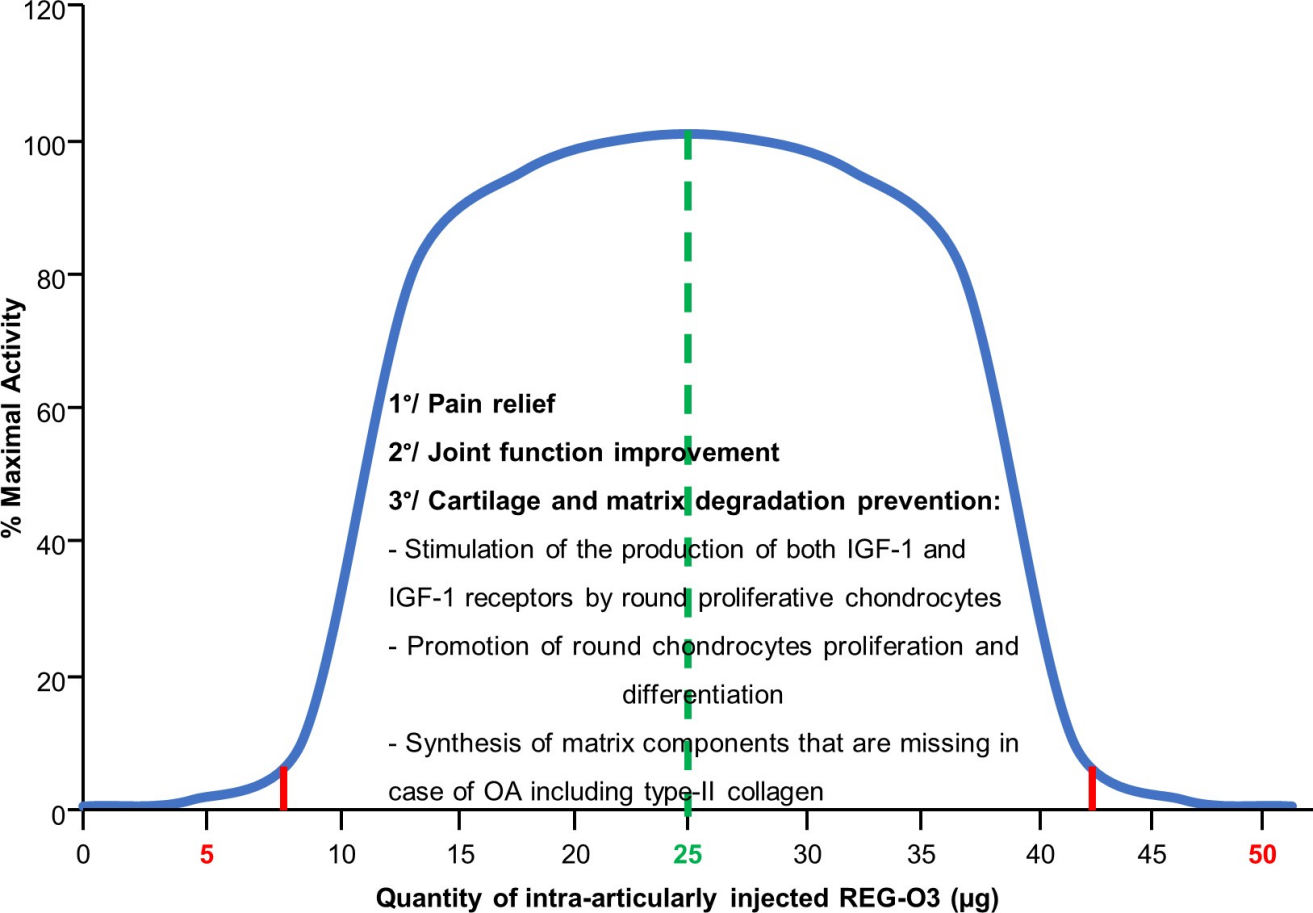
Working model for REG-O3 mechanism of action. Similar to GH, REG-O3 action displays a bell-shaped concentration dependence. When intra-articularly injected either at 5 μg or 50 μg, REG-O3 is inactive. The active range of intra-articularly injected REG-O3 spans from 7.5 μg to 42.5 μg where 25 μg represents the top of the bell.

GH exerts its biological effects *via* specific receptors binding [31]. Thus, GH administration shows classical dose-response effects until the saturation of receptors is reached [32]. Interestingly, it has also been demonstrated that at very high GH concentrations, the responses become dose dependently diminished. Thus, the GH action exhibits a bell-shaped concentration dependence rather than a simple sigmoid [33]. The IA injections of 5 μg and 50 μg of REG-O3 displayed both similar effects on pain/joint function and absence of effects on overall cartilage degeneration while the administration of 25 μg of REG-O3 showed higher effects. We thus propose that REG-O3 activity, which contains a GH fragment, displayed a bell-shaped concentration dependence in which the dose of 25 μg represents the top of the bell (Fig 8).

As a model for the protective effect on cartilage degradation, we hypothesize that REG-O3 modulates IGF-1-dependent signals with a fine tuning *via* its GH fragment. We propose that REG-O3 stimulates the production of both IGF-1 and IGF-1 receptors (IGF-1R) (patent WO 2010/105685 A) which then promotes the differentiation of round chondrocytes that synthetizes matrix components that are missing in case of OA including type-II collagen.

We therefore speculate that 25 μg of REG-O3 IA-injected in the knee of OA surgically-induced rats slowed down cartilage and matrix degradation through the GH-dependent local release of IGF-1 and type-II collagen production. By contrast, REG-O3 administered at either 5 μg or 50 μg is not able to induce GH-dependent local release of IGF-1 and type-II collagen associated production (Fig 8). This hypothetic mechanism should be investigated by measuring IGF-1 in synovial fluid of REG-O3-treated animals. Unfortunately, current methods do not allow such measurement in a so small volume as collected in rat. Another hypothesis is based on the presence of the GH fragment since it was demonstrated that intra-articular GH or GH analogs injection accelerate both healing and cartilage regeneration in animal models of OA [34–36]. Furthermore, a clinical study has shown that IA injections of GH improved pain and function of the osteoarthritic knee joint in a short period of time [14]. Then, we assume that the decreased pain and improved joint function we see in the REG-O3-treated rats is due to the synergic effect of both SST and GH fragments of REG-O3. We provide in Fig 8 a model for REG-O3 mechanism of action. It still has to be confirmed.

We have demonstrated here that REG-O3 was able to both improve joint function and protect cartilage from degeneration in rat with OA induced by anterior cruciate ligament transection and partial medial meniscectomy. Even if these results need to be confirmed in other animal models by studying other OA phenotypes, they remain remarkable compared to dexamethasone and hyaluronic acid. Thus, this study provides interesting data regarding both symptomatic and structural REG-O3 properties as a new Disease-Modifying OA Drug candidate, and therefore gives a rationale for testing this drug in Human. Collectively, the present work provides important information for the design of future clinical trials in terms of injected doses and timing of injections.

## Supporting information

S1 Appendix In vitro cytotoxicity assessment–micronuclei assay. (DOCX)

S2 Appendix REG-O3 stability in synovial fluid samples. (DOCX)

S1 Fig (a) and (b) Left knees histological score.
(TIF)

S2 Fig Right knees histological score: Non-significant results. (TIF)

S1 Dataset Complete study raw data. (XLSX)
