# Supplementary material for: REG-O3 chimeric peptide combining growth hormone and somatostatin sequences improves joint function and prevents cartilage degradation in rat model of traumatic knee osteoarthritis
Source: PLoS One. 2020 Apr 14;15(4):e0231240. doi: 10.1371/journal.pone.0231240 (PMC7156079; doi:10.1371/journal.pone.0231240)
Supplement: S1 Appendix — (DOCX) [file pone.0231240.s001.docx]

***In vitro* cytotoxicity assessment – Micronuclei assay**

**Method**

V79-4 cell lines were grown as described by the supplier (ATCC^®^ CCL-93^™^). Those cells, which show a doubling time of 12-14h, were treated continuously with the items in duplicate during 24h (long treatment) according to the OECD 487 guideline. Of note, as positive controls, cells were incubated with either mitomycin (clastogen) or colchicine (aneugen). As a negative control, cells were grown with the vehicle (PBS, untreated).

The cytotoxicity/genotoxicity evaluation was based on the cytokinesis-block proliferation index (CBPI) quantification, replication index (RI) and micronuclei induction measurement. The CBPI represents the proportion of cells from the second division in the treated population compared to the untreated control and indicates the mean number of nuclei per cell. More than 1000 cells were counted in each group of treatment and the CBPI was calculated this way:

((nb of mononucleated cells) + (2 × nb of binucleated cells) + (3 × nb of multinucleated cells))

**CBPI** =

(total number of cells)

The RI indicates the relative number of cell cycles per cell performed during the period of exposure in the treated cultures compared to the control cultures. More than 1000 cells were counted in each group of treatment and the RI was calculated this way:

((nb of binucleated treated cells) + (2 × nb of multinucleated treated cells)) ÷ (total number of treated cells)

**RI** = × 100

((nb of binucleated untreated cells) + (2 × nb of multinucleated untreated cells)) ÷ (total number of untreated cells)

Of note, for the micronuclei induction evaluation, 2000 cells were counted. The test is considered valid if the level of micronuclei in the negative control is between 5 and 25 micronuclei per 1000 cells counted.

**Results**

We first studied the putative cytotoxic properties of our REG-O3 compound. To this end, the V79-4 cell lines were grown in the presence of increasing concentrations of REG-O3 during twice the doubling time of such cell lines i.e 24h. Even at 1.8-time the highest final concentration (178 µM) we have tested in the rat (50 µg or 100 µM), the cells treated with either REG-O3 or the negative control item (PBS) displayed similar CBPI and RI. Indeed, the control cells exhibited a mean CBPI of 1.57 whereas the cells grown with the different concentrations of REG-O3 showed a mean CBPI varying from 1.56 to 1.60 (Fig S1A). On the other hand, regarding the RI, while the negative control presented a mean percentage of 104%, the cells incubated with the increasing concentrations of REG-O3 displayed a mean RI percentage varying from 102.9% to 108.8% (Fig S1B). Of note, the cells treated with the colchicine positive control item exhibited a decrease of the RI percentage to 92.7% (Fig S1B). In total, in these experimental conditions, REG-O3 did not induce *in vitro* cytotoxic effects.


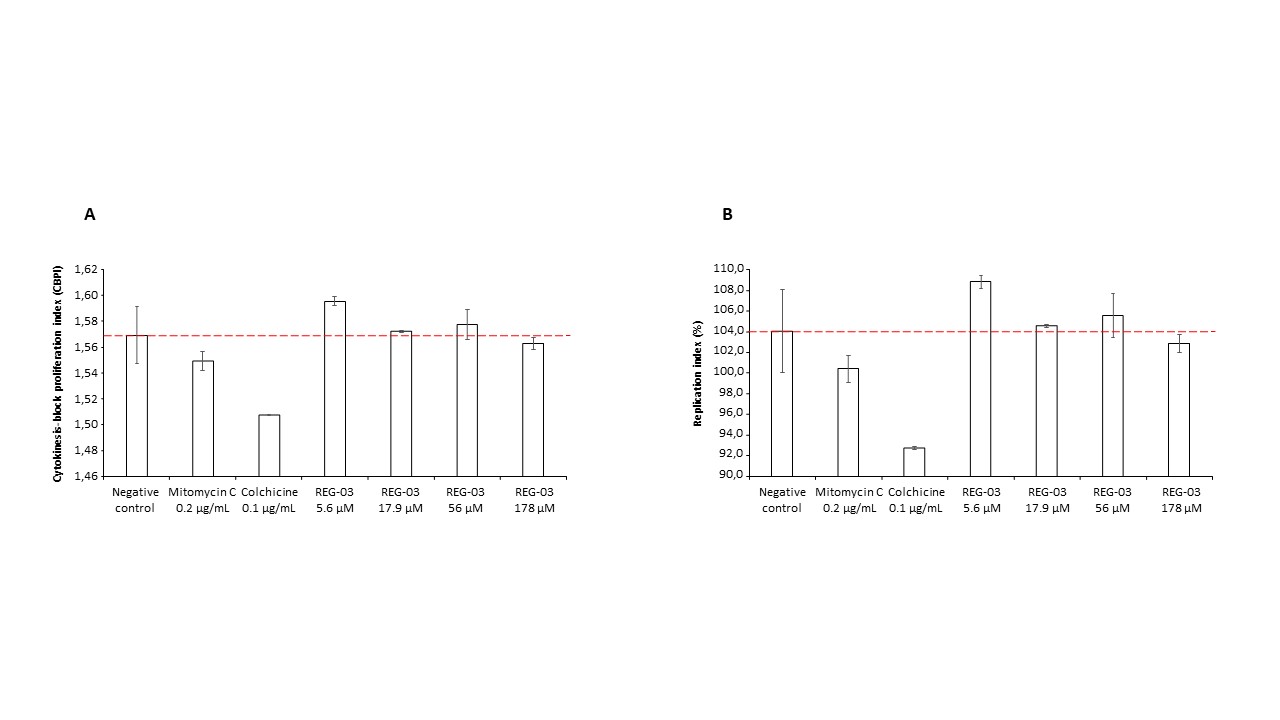


**Fig S1 : In vitro cytotoxicity assessment upon a 24h-treatment in V79-4 cell lines.**

**A.** Cytokinesis-block proliferation index (CBPI) **B.** Replication index (RI). These indices were quantified upon incubation with either positive control items regarding cytotoxicity (mitomycin and colchicine) or increasing concentrations of REG-O3. Data are presented as the mean +/- sem from 2 independent experiments. In total, more than 1000 cells were counted in each experimental condition.

The putative genotoxic property of REG-O3 was analysed by counting the number of cells with micronuclei upon a 24h-treatment. The assay was considered as valid if both the percentage of micronuclei was comprised between 0.5% and 2.5% for the cells treated with the vehicle and the positive control items showed a clear positive effect with a percentage of cells with micronuclei >2%.

In the present case, while 1.15% of the vehicle-incubated V79-4 cells displayed micronuclei, 3.55% of the mitomycin-treated cells and 3.85% of the cells grown in the presence of colchicine did (Fig S2). The assay was therefore valid. We then examined the percentage of cells with nuclei upon REG-O3 treatment. Whatever the concentration of REG-O3 tested, the percentage of cells with micronuclei was lower than 2%. Even at 1.8-time the highest final concentration (178 µM) we have tested in the rat (50 µg or 100 µM), only 1.6% of the cells treated with REG-O3 presented micronuclei (Fig S2). In these experimental conditions, REG-O3 did not induce micronuclei i.e. *in vitro* genotoxic effects.


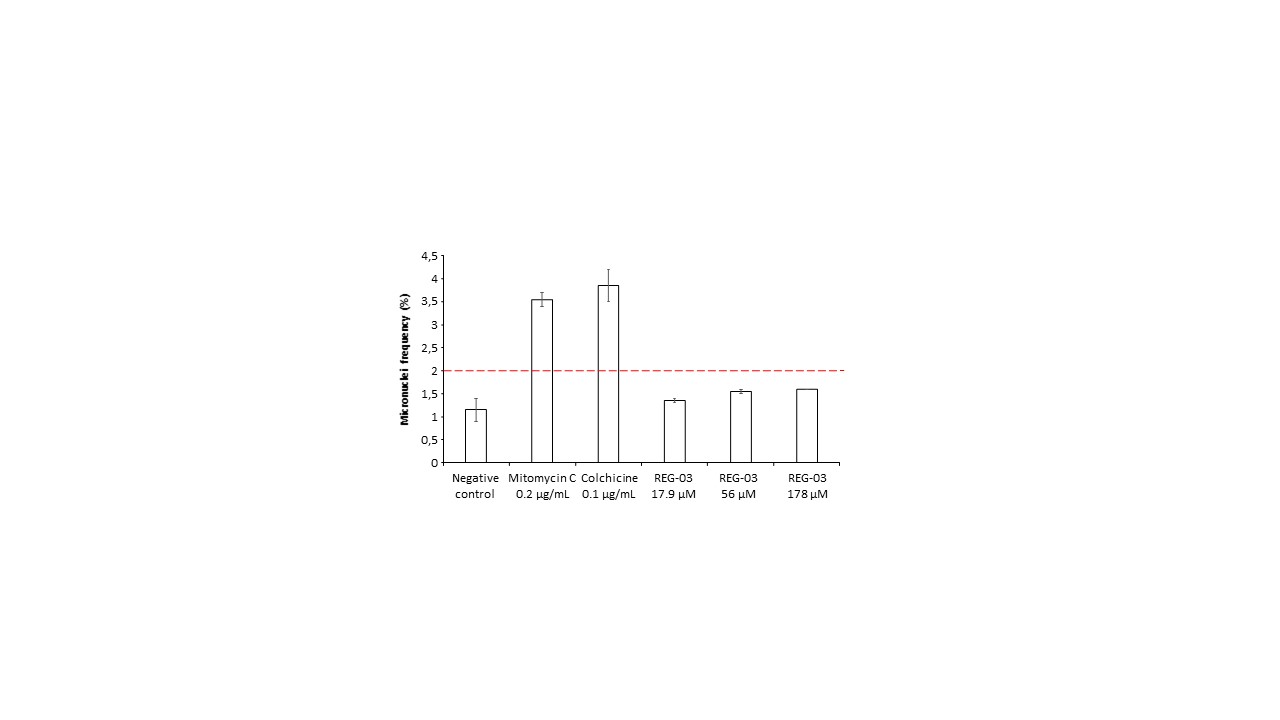


.

**Fig S2 : In vitro genotoxicity assessment (percentage of micronuclei) upon a 24h-treatment in V79-4 cell lines.**

These percentages were quantified upon incubation with either positive control items regarding genotoxicity (mitomycin and colchicine) or increasing concentrations of REG-O3. Data are presented as the mean +/- sem from 2 independent experiments. In total, 2000 cells were counted in each experimental condition. Red line shows the threshold for genotoxic effect.
