# Supplementary material for: REG-O3 chimeric peptide combining growth hormone and somatostatin sequences improves joint function and prevents cartilage degradation in rat model of traumatic knee osteoarthritis
Source: PLoS One. 2020 Apr 14;15(4):e0231240. doi: 10.1371/journal.pone.0231240 (PMC7156079; doi:10.1371/journal.pone.0231240)
Supplement: S2 Appendix — (DOCX) [file pone.0231240.s002.docx]

**REG-O3 stability in synovial fluid samples**

**Method**

1. ***Human synovial fluid collection and preparation***

For this study, a large volume of synovial fluid was needed. Since we were unable to collect more than 200 µL of synovial fluid in rats, we decided to collect such samples from Human patients who underwent synovial fluid punctures upon knee swelling. These samples were obtained *via* Dr. Andrea Baldini (IFCA Clinic, Villa Ulivella, Florence, Italy) with patients’ consent. Samples were collected in sterile tubes cooled at 4°C for 10 min and then centrifuged at 3000 × g for 10 min, to remove cells and joint debris. The centrifugate synovial fluid was diluted to 50% in PBS and dosed using Bradford assay. 1 mL of the diluted synovial fluid (500 µL of PBS + 500 µL of synovial fluid) was temperature-equilibrate at 37±1°C for 15 min.

1. ***REG-O3 peptide preparation***

REG-O3 stock solution was prepared at 5 mg/mL in 50% DMSO. This solution was aliquoted and stored at -20°C.

1. ***Addition of REG-O3 peptide in Human synovial fluid and collection of sample solution reaction***

The REG-O3 stock solution was thawed and maintained at 4°C before use. 50 μL of this peptide solution was added in the 1 mL of diluted synovial fluid generated above that has been temperature-equilibrate at 37±1°C. At known time intervals (0, 2h, 4h, 6h, 24h, 30h, 48h), 100 µL of the reaction solution was collected.

1. ***Analysis of peptide degradation – Determination of half-life***

Once those 100 µL collected, 200 µL of ethanol were added to the solution containing both REG-O3 and the Human synovial fluid. The cloudy reaction sample was cooled at 4°C for 15 min and then spun at 11.000 rpm for 2 min to pellet the precipitated protein.

The supernatant was analysed by a reverse-phase High Performance Liquid Chromatography (HPLC) method that separated the parent peptide from its degradation products. The products were characterised by reverse-phase HPLC. Products were analysed and characterised by ACQUITY HPLC (thermo finnigan surveyor autosampler) coupled to UV (215 nm) Thermo Finnigan Surveyor PDA Detector and thermo finnigan lcq advantage using a C18 Phenomenex Aqua (250 x 4,6 mm, 5 μ, C18, 300Å) at 35°C, with a flow rate of 0.6 mL/min. The solvent systems used were A (0.1% TFA (TriFluoroAcetic acid) in H_2_O) and B (0.1% TFA in acetonitrile). The supernatant was analysed by a reverse-phase HPLC method (gradient from 10% to 90% of acetonitrile in water, 20 min, 1 mL/min, 20 µL injection). REG-O3 and its degradation products were identified by mass spectrometry.

**Results**

We studied the stability of the REG-O3 peptide in synovial fluids from nine control patients (non-affected by gonarthrosis) and ten patients suffering from gonarthrosis (Fig S3A). Overall, whatever the study group, native REG-O3 was still detectable in synovial fluids after 24h-30h (Fig S3A).

To be more specific, in synovial fluids from control patients, REG-O3 was detectable after 24 hours of incubation in 3 samples, 30 hours in 5 samples and 6 hours in 1 sample (Fig S3A). In synovial fluids from patients affected by gonarthrosis, REG-O3 was detectable after 24 hours of incubation in 8 samples, 30 hours in 1 sample and 6 hours in 1 sample (Fig S3A).

In total, REG-O3 was still detectable after 25.3 hours of incubation in synovial fluids from control patients whereas it remained detectable after 22.8 hours in synovial fluids from patients with gonarthrosis. Interestingly, statistical analyses showed that this slight difference was not significant (Fig S3B). This strongly suggests that, whatever the patient status (affected or not by both gonarthrosis and knee inflammation), REG-O3 presented a prolonged stability in synovial fluid.


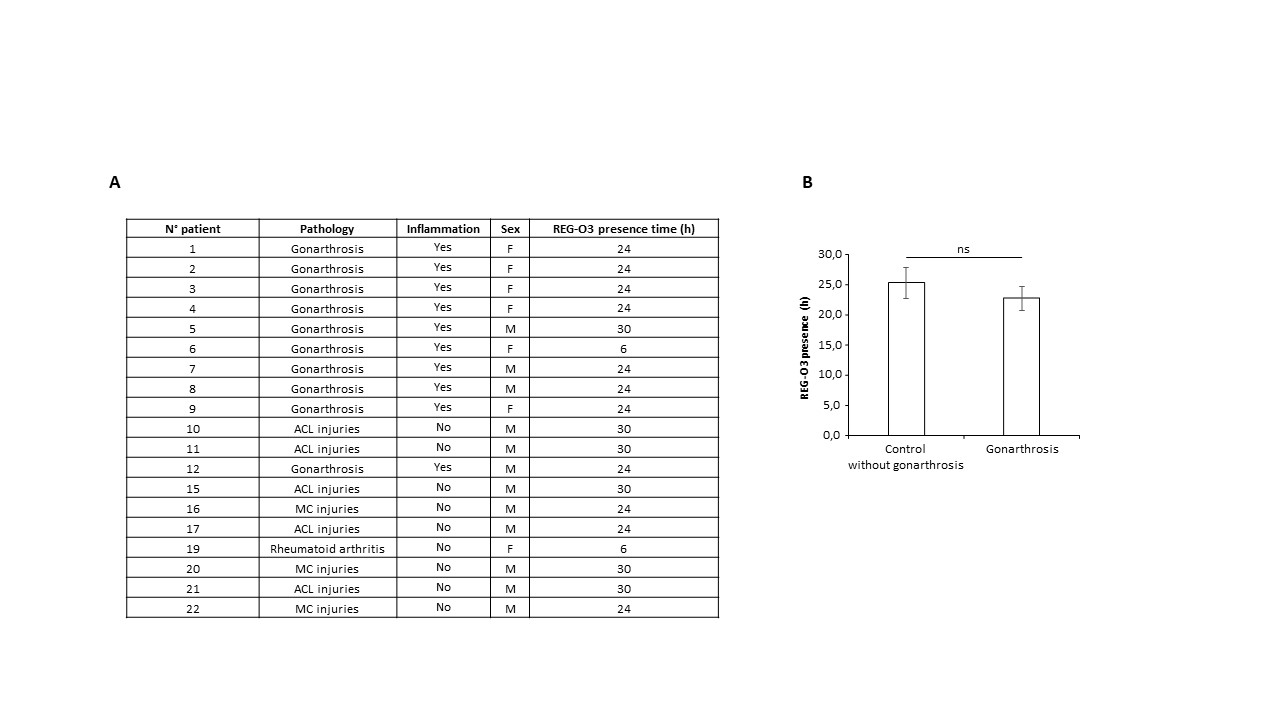


**Fig S3 : REG-O3 presence time in synovial fluids from Human patients.**

**A.** Table presenting the information relative to the collected samples including REG-O3 presence time in each of them (MC=meniscus, ACL= Anterior Cruciate Ligament). **B.** The REG-O3 presence time in each type of synovial fluid. Data are presented as the mean +/- sem. Data were compared using a Mann-Whitney U-test for non-parametric values (ns: p=0.0837 was considered as non-significant).
