# Supplementary figures and images for: REG-O3 chimeric peptide combining growth hormone and somatostatin sequences improves joint function and prevents cartilage degradation in rat model of traumatic knee osteoarthritis
Source: PLoS One. 2020 Apr 14;15(4):e0231240. doi: 10.1371/journal.pone.0231240 (PMC7156079; doi:10.1371/journal.pone.0231240)

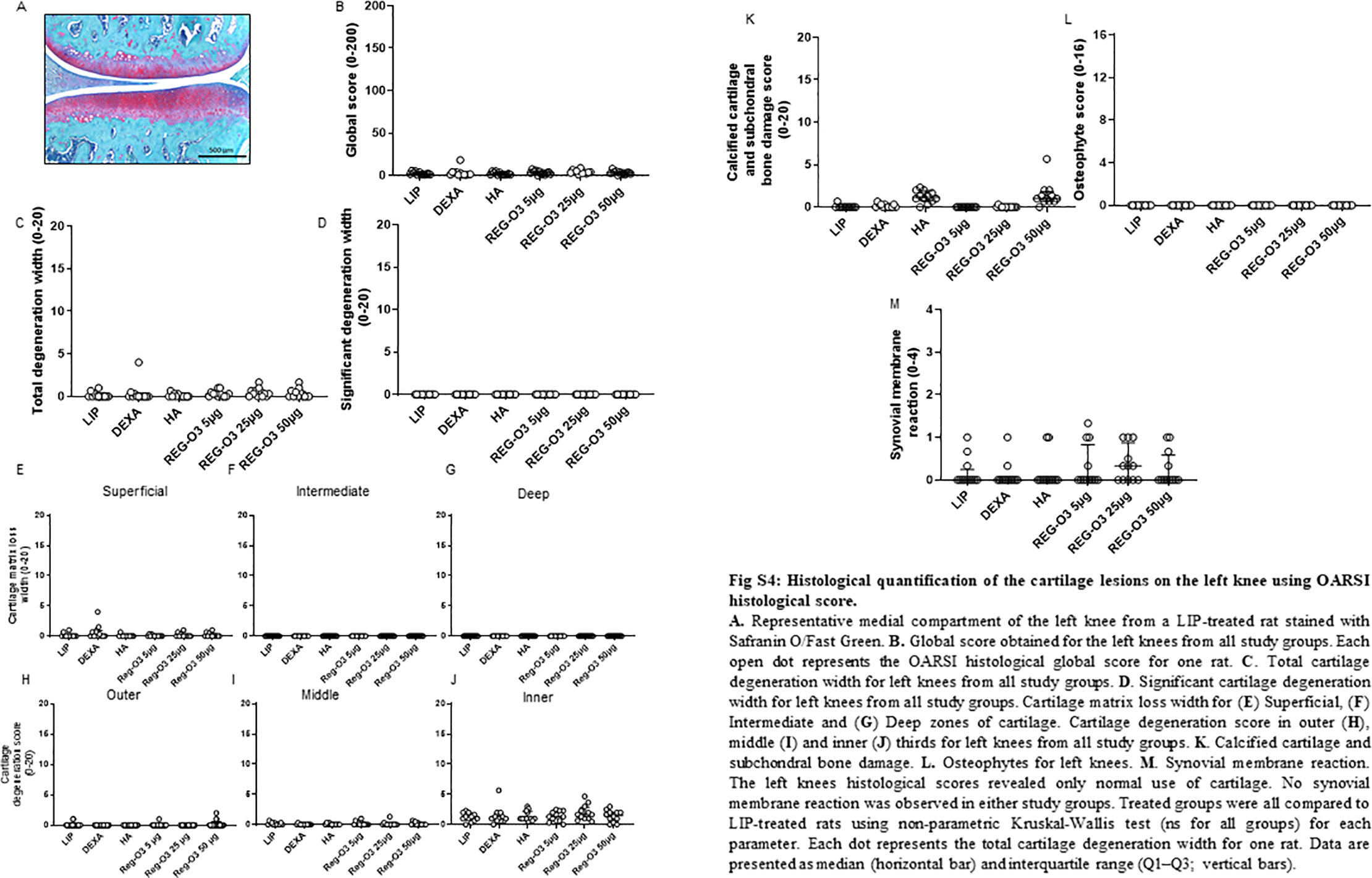

Supplement: S1 Fig — (a) and (b) Left knees histological score. (TIF) [file pone.0231240.s003.tif]

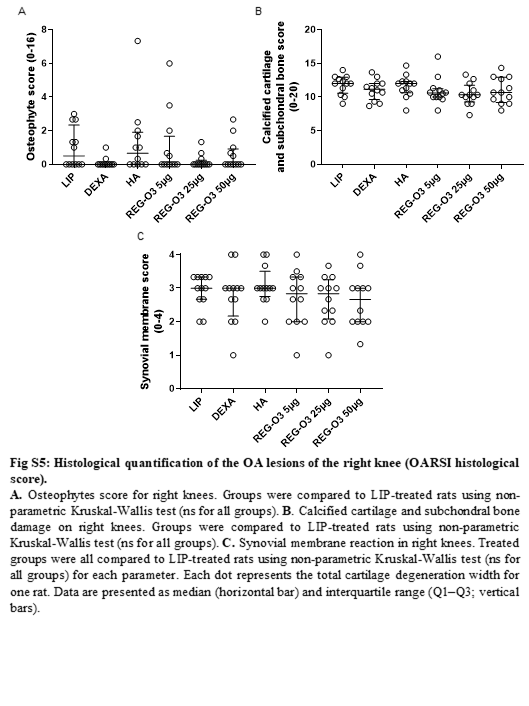

Supplement: S2 Fig — (TIF) [file pone.0231240.s004.tif]
